# Exploring Medicinal Herbs’ Therapeutic Potential and Molecular Docking Analysis for Compounds as Potential Inhibitors of Human Acetylcholinesterase in Alzheimer’s Disease Treatment

**DOI:** 10.3390/medicina59101812

**Published:** 2023-10-12

**Authors:** Ayoub Farihi, Mohamed Bouhrim, Fatiha Chigr, Amine Elbouzidi, Noureddine Bencheikh, Hassan Zrouri, Fahd A. Nasr, Mohammad Khalid Parvez, Ahmad Alahdab, Ahmed Omar Touhami Ahami

**Affiliations:** 1 Unit of Clinic and Cognitive Neuroscience, Laboratory of Biology and Health, Department of Biology, Faculty of Sciences, Ibn Tofail University, Kenitra 14000, Morocco; ayoub.farihi@uit.ac.ma (A.F.); ahami_40@yahoo.fr (A.O.T.A.); 2 Bioengineering Laboratory, Faculty of Sciences and Techniques, Sultan Moulay Slimane University, Beni Mellal 23000, Morocco; mohamed.bouhrim@gmail.com (M.B.); f.chigr@usms.ma (F.C.); bencheikh_noureddine1718@ump.ac.ma (N.B.); 3 Laboratory for Agricultural Production Improvement, Biotechnology, and Environment (LAPABE), Faculty of Science, Mohammed First University, Oujda 60000, Morocco; 4 Laboratory of Bioresources, Biotechnology, Ethnopharmacology and Health, Faculty of Sciences, Mohammed First University, Boulevard Mohamed VI, B.P. 717, Oujda 60000, Morocco; hzrouri@yahoo.fr; 5 Department of Pharmacognosy, College of Pharmacy, King Saud University, Riyadh 11451, Saudi Arabia; fnasr@ksu.edu.sa (F.A.N.); mohkhalid@ksu.edu.sa (M.K.P.); 6 Institute of Pharmacy, Clinical Pharmacy, University of Greifswald, Friedrich-Ludwig-Jahn-Street 17, 17489 Greifswald, Germany

**Keywords:** Alzheimer’s disease, phytochemicals, medicinal herbs, molecular docking, ADMET analysis, acetylcholinesterase inhibitors

## Abstract

*Background and Objectives*: Alzheimer’s disease (AD) stands as a pervasive neurodegenerative ailment of global concern, necessitating a relentless pursuit of remedies. This study aims to furnish a comprehensive exposition, delving into the intricate mechanistic actions of medicinal herbs and phytochemicals. Furthermore, we assess the potential of these compounds in inhibiting human acetylcholinesterase through molecular docking, presenting encouraging avenues for AD therapeutics. *Materials and Methods*: Our approach entailed a systematic exploration of phytochemicals like curcumin, gedunin, quercetin, resveratrol, nobiletin, fisetin, and berberine, targeting their capability as human acetylcholinesterase (AChE) inhibitors, leveraging the PubChem database. Diverse bioinformatics techniques were harnessed to scrutinize molecular docking, ADMET (absorption, distribution, metabolism, excretion, and toxicity), and adherence to Lipinski’s rule of five. *Results:* Results notably underscored the substantial binding affinities of all ligands with specific amino acid residues within AChE. Remarkably, gedunin exhibited a superior binding affinity (−8.7 kcal/mol) compared to the reference standard. *Conclusions:* These outcomes accentuate the potential of these seven compounds as viable candidates for oral medication in AD treatment. Notably, both resveratrol and berberine demonstrated the capacity to traverse the blood-brain barrier (BBB), signaling their aptitude for central nervous system targeting. Consequently, these seven molecules are considered orally druggable, potentially surpassing the efficacy of the conventional drug, donepezil, in managing neurodegenerative disorders.

## 1. Introduction

Alzheimer’s disease (AD), a prevalent neurodegenerative disorder, is commonly known as senile dementia and is characterized by cognitive decline and memory loss [1,2]. It is the most prevalent form of dementia in the aging population [3], leading to irreversible damage in various brain regions [4]. The global number of AD patients was estimated to be nearly 50 million in 2018, with projections suggesting that this number could reach 150 million by 2050 [5]. While AD is recognized as a complex disease influenced by both environmental and genetic factors, including gender and family history, its exact etiology remains uncertain. Despite well-identified histopathological features within the brain, the biochemical mechanisms of AD are not yet fully understood [6]. The prevailing amyloid hypothesis has garnered substantial support, suggesting that AD is a progressive and irreversible neurodegenerative disease characterized by the aggregation of microtubule-associated protein tau into neurofibrillary tangles (NFTs) within neurons and the extraneuronal deposition of amyloid β (Aβ) protein as senile plaques. Aβ is formed through the sequential proteolytic cleavage of amyloid precursor protein (APP) by γ- and β-secretases in the amyloidogenic pathway [2,7]. Accumulation of Aβ results in the enlargement of senile amyloid plaques, starting from allocortical and limbic regions and eventually impacting the hippocampus and entorhinal cortex [8,9]. The presence of Aβ disrupts synaptic signaling pathways, resulting in the impairment of behavior and memory through the destruction of dendritic spines and alterations in synaptic morphology [10,11]. Imaging methods depend on detecting amyloid deposits as part of the distinctive diagnosis process for AD [12,13]. Contemporary research is primarily centered on Aβ as a target for creating therapeutic medications, such as anti-Aβ antibodies and vaccines [11,14]. Nevertheless, options for treating AD symptoms remain restricted, and there is a notable absence of effective therapies to decelerate disease progression. Researchers are currently exploring traditional medicinal plants, historically used to address memory-related disorders, as potential sources for novel treatments for AD [15].

One of the major contributors to AD is the decreased levels of the neurotransmitter acetylcholine (ACh) at synapses in the human cerebral cortex, leading to impaired cognitive functions and memory loss [16]. Reactive oxygen species (ROS), generated during metabolic processes, contribute to oxidative stress, which is implicated in the development of degenerative diseases like AD [17]. Natural antioxidants, such as flavonoids and polyphenols, function by neutralizing ROS, especially free radicals, by providing them with an extra electron. This process helps stabilize these highly reactive molecules, thus reducing oxidative damage in the brain [18].

Multiple studies have demonstrated that the neurotransmitter acetylcholine (ACh) levels decrease due to reduced choline (Ch) and acetyltransferase activity, leading researchers to focus on cholinesterase (ChE) inhibitors as a symptomatic treatment option. By inhibiting the cholinesterase enzymes, including butyrylcholinesterase (BChE) and acetylcholinesterase (AChE), it may be possible to restore ACh levels, thereby improving cholinergic transmission and reducing amyloid aggregation, which could potentially benefit individuals with dementia and Alzheimer’s disease [3,19]. Consequently, these studies have revealed the AChE inhibitory effects of active natural compounds found in plant extracts. Several plants contain secondary metabolites with antioxidant and anti-AChE properties, which can slow the progression of Alzheimer’s disease by inhibiting AChE activity and protecting neurons from oxidative damage [4,5,20,21]. Numerous phytochemicals and herbs have been investigated for their therapeutic potential in Alzheimer’s disease, and significant progress has been made in this area. To compile this article, we conducted an extensive search of the Scopus and MEDLINE (PubMed) databases, utilizing relevant terms such as phytomedicine, herb, phytochemical, and Alzheimer’s. Our objective is to provide a comprehensive summary of the current understanding of the effects of phytochemicals and herbs contained in their extract molecules which exert a neuroprotective effect, which plays a fundamental role in the action of plant extracts against Alzheimer’s disease. Moreover, this research aimed to elucidate the binding affinity and interaction between acetylcholinesterase and various compounds through the use of molecular docking. The utilization of these widely available and easily accessible plants as sources of valuable phytochemicals for the pharmaceutical industry is an area of great interest [3,4,5,19,20,21].

## 2. Medicinal Herbs against Alzheimer’s

Phytotherapy, also known as Herbalism in Western medicine, is a specialized branch of medicine that harnesses the therapeutic potential of plants and natural active ingredients for the treatment of diseases or as agents to promote overall health [22]. In the context of Alzheimer’s disease, herbs have emerged as potential candidates for therapeutic intervention and may hold promise for the development of effective natural anti-Alzheimer’s drugs in the future [23].

### 2.1. Rosa damascena Herrm.

*R. damascena* is part of the family Rosaceae [24], a plant used in traditional medicine [25]. It contains bioactive secondary metabolites, including anthocyanins, with pelargonidin 3-glucoside as the predominant compound, constituting 80.6% of all anthocyanins [26]. Cerezo et al. demonstrated that pelargonidin 3-glucoside possesses antioxidant activity [27]. It contains phenolic compounds such as quercetin and kaempferol [26], which have a beneficial effect on the central nervous system [28,29]. In rats, Esfandiary et al. showed that *R. damascena* extract (300, 600, 1200 mg/kg p.o for 1 month) reversed behavioral deficits caused by Aβ in AD [30]. It increased the transcription factor CREBR, the expression of the neurotrophic factor, and the both the total volume of hippocampus and the absolute volume (CA1, DG), which were altered under the influence of Aβ [31].

### 2.2. Picrasma quassioides (D.Don) Benn.

*Picrasma quassioides* belongs to the family Simaroubaceae, mostly distributed in the temperate to tropical areas of East Asia, including China and Japan [32]. *P. quassioides* possesses many medicinal properties, including anti-inflammatory [33], antimalarial, antihypertensive, antibacterial, anticancer, antioxidant effects [34,35,36], as well as neuroprotective activity, potency as gastric indigestion and asthma treatments, and as a preventative treatment for osteoporosis [37]. Guo et al. showed that compounds in this plant have exhibited neuroprotective activities. In mice, *P. quassioides* extract (25, 50, and 100 mg/kg, p.o) inducted a neuroprotective activity in Aβ25-35-stimulated SH-SY5Y and L-glutamate-stimulated PC12 cell models along with improved cognitive abilities and memory in AD mice induced by Aβ [38].

### 2.3. Actinidia arguta (Siebold & Zucc.) Planch. ex Miq.

*Actinidia arguta*, a plant belonging to the Actinidiaceae family, has been traditionally used in Korea for treating inflammatory and gastrointestinal diseases [39]. Moreover, research has revealed several beneficial properties associated with *A. arguta*, including anti-inflammatory, antiapoptotic, antioxidant, and anti-allergic effects [40,41]. It is also known to contain numerous antioxidants such as catechins, anthocyanin, carotenoids, chlorophyll, and vitamin C [42,43]. It contains phenolic compounds such as quercetin and kaempferol [44], which have a beneficial effect on the central nervous system [28,29]. In a study conducted on mice by Su Ha et al., it was found that the extract of *A. arguta* at different doses (5, 10, and 20 mg/kg, orally) significantly improved memory and learning deficits induced by Aβ (amyloid-beta). Additionally, the extract exhibited beneficial effects on cholinergic functions and protected antioxidant systems. This was achieved by reducing AChE activity, increasing ACh levels, lowering oxidized glutathione (GSH)/total GSH ratio and malondialdehyde (MDA) levels, and increasing superoxide dismutase (SOD) levels in the brain. Furthermore, *A. arguta* extract was found to prevent mitochondrial dysfunction by normalizing levels of apoptotic signaling molecules, including phosphorylated tau (p-tau), cytochrome c, and phosphorylated Akt (p-Akt) in the brain tissues [45].

### 2.4. Alpinia galanga *(L.)*

*Alpinia galanga* (Zingiberaceae) is generally dispersed in India. It is an aromatic perennial that resembles a rhizome, is traditionally used as a nerve tonic and stimulant. It is still used as a repellent, digestive aid, aphrodisiac, stomach tonic, anti-inflammatory, and antiseptic [46]. Many molecules are isolated and tested for biological activity, including terpenyl ester (2-endohydroxy-1,8-cineole) for antibacterial activity and antimicrobial [47], essential oils with hypoglycemic activity [48], antifungal activity [49], and in vitro cholinesterase enzyme inhibition [50]. It contains phenolic compounds such as quercetin and kaempferol [51], which have a beneficial effect on the central nervous system [28,29]. In mice, Singh et al. showed that Alpinia galanga extract (200 and 400 mg/kg, oral administration) induced increases in Na+/K+-ATPase, free radical scavenging property and improved brain membrane integrity [46].

### 2.5. Piper nigrum *L.*

*P. nigrum* is a common plant in the Piperaceae family. It is cultivated in Pakistan, South America, Africa, and the southwestern Indian highlands. *P. nigrum*’s main alkaloid component is piperine. The spiciness and pungency of black pepper is due to the alkaloidal elements found in the fruit. It is used as a spice all over the world, and since it contains pharmacological characteristics, it is utilized in ancient medicinal systems like Ayurveda and Unani medicine to treat diseases, pain, fever, and inflammation [52]. It contains limonene [53], a molecule with neuroprotective effects [54]. In rats, Hritcu et al., 2014, showed that *P. nigrum* extract (50 and 100 mg/kg, p.o) inhibiting oxidative stress in the hippocampus, which ameliorates β-amyloid (1–42)-induced spatial memory impairment [55].

### 2.6. Rheum ribes *L.*

*R. ribes* is a plant that is part of the family of Polygonaceae, which is generally used in traditional remedies due to its many biological activities, including antibacterial and antioxidant [56]. It contains curcumin [57], a molecule with neuroprotective effects [58]. Yildirim et al. showed that *R. ribes* extract increased antioxidant enzyme activities and diminished blood glucose levels in STZ-induced diabetic rats [59]. Zahedia et al. showed that *R. ribes* extract (250 and 500 mg/kg, p.o) ameliorates memory deficits generated by bilateral NBM lesions in rats [60].

### 2.7. Markhamia tomentosa (Benth.) K. Schum. ex Engl.

*M. tomentosa*, a plant belonging to the Bignoniaceae family [61], has been found to be safe for oral administration in rats, as demonstrated by Ibrahim et al. This indicates a lack of toxicity on renal and hepatic function parameters [62]. The extract of *M. tomentosa* has been reported to possess various beneficial properties, including antioxidant, antimicrobial, anti-inflammatory, antiulcer, and analgesic activities [63,64,65,66]. It contains catechin [67], a molecule with neuroprotective effects [68]. In a separate study conducted by Ionita et al. on rats, the oral administration of *M. tomentosa* extract at doses of 50 and 200 mg/kg resulted in improved memory performance in behavioral tests. Additionally, the extract exhibited anti-acetylcholinesterase activity and reduced oxidative stress in the rat hippocampus [67].

### 2.8. Cassia obtusifolia *L.*

*C. obtusifolia*, a plant belonging to the Leguminosae family, is commonly used in traditional Chinese medicine. The seeds of this plant, known as Juemingzi in Chinese, have a history of being utilized to address various conditions, such as red and watery eyes, dizziness, and headaches [69]. Researchers have extensively investigated the chemical composition of these seeds, leading to the isolation of numerous anthraquinones [70]. It contains emodin [71], a molecule with neuroprotective effects [72]. In a study involving mice, Kim et al. demonstrated that the oral administration of *C. obtusifolia* extract at doses of 25, 50, and 100 mg/kg resulted in the alleviation of memory impairment induced by scopolamine. Additionally, the extract inhibited acetylcholinesterase, leading to an improvement in the cholinergic function of the nervous system [73].

## 3. Phytochemicals against Alzheimer’s Disease

Curcumin, resveratrol, nobiletin, berberines, limonoid, galantamine, and quercetin are a group of naturally occurring phytochemicals derived from various plants. Extensive research has demonstrated their potential to reduce the risk of various debilitating conditions, including neurodegenerative diseases, cardiovascular diseases, depression, and diabetes. Notably, these phytochemicals exhibit neuroprotective properties, suggesting their capacity to mitigate the symptoms associated with Alzheimer’s disease.

### 3.1. Curcumin

Curcumin is a dietary polyphenol derived from *Curcuma longa* L. [74], a spice commonly used in India, Southeast Asia, and China for its aromatic, coloring, and preservative properties [75]. Extensive research has elucidated the molecular mechanisms underlying the therapeutic effects of curcumin, including its anticancer, antioxidant, anti-inflammatory, antidiabetic, immunomodulatory, lipid-regulating, hepatoprotective, antiarthritic, antidepressant, and anti-Alzheimer’s properties [74,76]. Despite its broad range of effects, curcumin possesses intrinsic physicochemical characteristics such as photodegradation, low bioavailability, poor water solubility, short half-life, and chemical instability [77,78].

The anti-Alzheimer’s effects of curcumin have been demonstrated in various murine models. In mice, intraperitoneal administration of curcumin at a dosage of 50 mg/kg resulted in a reduction of Aβ plaque burden in the dentate gyrus of the hippocampus and prefrontal cortex (PFC). Moreover, a significant decrease in pyknotic or tangle-like neurons was observed in regions such as CA1 and CA3 of the hippocampus and the PFC, along with a decrease in the expression of Iba-1 and GFAP in the PFC [79]. Ray et al. reported that intraperitoneal injection of curcumin at a dosage of 25 mg/kg led to a decrease in H_2_O_2_ levels and an increase in glutathione (GSH) levels in the brains of athymic mice, suggesting a favorable intracellular redox environment. Additionally, the increased ratio of free to oxidized glutathione (GSH: GSSH) indicated an improved redox status compared to controls [80]. In rats, Doaa et al. demonstrated that oral administration of curcumin at a dosage of 80 mg/kg reduced β-amyloid accumulation in the hippocampus and ameliorated cognitive deficits [81]. Han-chang et al. found that intraperitoneal injection of curcumin at a dosage of 10 mg/kg reduced oxidative stress, improved active avoidance and locomotor activity, and reduced neurodegeneration in experimental models [82].

### 3.2. Gedunin

Gedunin is a principal limonoid found primarily in the seeds of many genera of the family *Meliaceae*. Many biological activities have been attributed to gedunin, such as antimalarial, anticancer, antiallergic, neuroprotective, antibacterial, anti-inflammatory, and insecticidal effects [83,84,85]. Thom et al. showed that gedunin inhibited the activation of NF-κB induced by Aβ1–42, thereby reducing the levels of nitric oxide (NO) and interleukin-1 beta (IL-1β), pro-inflammatory molecules. Furthermore, gedunin inhibits neuroinflammation by activating nuclear factor erythroid 2-related factor 2 (Nrf2) and its downstream targets, including γ-glutamylcysteine synthetase, heme oxygenase 1, and NADPH quinone dehydrogenase 1, which are involved in quenching reactive oxygen and nitrogen species (NO) generated by NF-κB activation [86].

### 3.3. Quercetin

Quercetin, a bioactive compound found in various vegetables and fruits, such as white onion bulbs, blueberries, and cranberries [87,88], exhibits distinct actions in the brain, influencing glucose homeostasis in experimental diabetes and conferring beneficial effects [89]. It possesses a multitude of health benefits, including antioxidant, anti-inflammatory, cardiovascular, anticancer properties, and neuroprotection [90,91,92,93,94,95]. Particularly in Alzheimer’s disease (AD), pretreatment of neuronal cultures obtained from 18-day-old Sprague–Dawley rat fetuses with quercetin significantly attenuated Aβ1–42-induced cytotoxicity at the two lowest doses (5 and 10μM), protein oxidation, lipid peroxidation and apoptosis [96]. In a study conducted by Manouchehr et al., rats treated with quercetin via intraperitoneal injection at doses of 40 and 80 mg/kg exhibited improved spatial memory in an AD model induced by intracerebroventricular administration of streptozotocin (ICV-STZ) [97].

### 3.4. Resveratrol

Resveratrol, a member of the polyphenol group of phytochemicals, is naturally present in wild fruits such as grapes and blueberries, offering beneficial effects on human health [98,99]. Notably, resveratrol has shown potential in improving cognitive function in dementia and playing a neuroprotective role in the neurodegenerative processes associated with Alzheimer’s disease [100]. It possesses antioxidant properties, making it potentially valuable in combating oxidative stress [101]. Additionally, resveratrol exhibits anticarcinogenic and anti-inflammatory properties [102]. In a study conducted by Yazir et al., rats treated with resveratrol via intraperitoneal injection at doses of 5 or 20 mg/kg for 35 days demonstrated a significant attenuation of scopolamine-induced deficits in spatial memory and emotional learning in chronically stressed rats [103]. Another study by Sharma and Gupta, revealed that intraperitoneal administration of resveratrol at doses of 10 and 20 mg/kg in rats significantly suppressed cognitive impairment induced by intracerebroventricular administration of streptozotocin (ICV-STZ). The resveratrol-treated ICV-STZ rats exhibited increased brain glutathione levels and a modest elevation in brain malondialdehyde levels [104].

### 3.5. Nobiletin

Nobiletin is an important polymethoxyflavones existing in fruits including oranges, tangerines, and lemons [105]. Diverse pharmacological effects attributed to nobiletin comprise antidiabetic, anti-atherogenic, antioxidant, anticarcinogenic and anti-inflammatory effects [106,107]. In rats, Matsuzaki et al. showed that nobiletin (50 mg/kg, intraperitoneally injected) acts as a protector against Aβ1–40-induced impairment of learning ability [108]. In mice, Akira Nakajima et al. showed that nobiletin (30 mg/kg, intraperitoneally injected for 3 months) ameliorates memory impairment in olfactory-bulbectomized mice and learning, NMDA receptor antagonist-treated mice and amyloid precursor protein transgenic mice. In the hippocampus, nobiletin reduced ROS levels [109].

### 3.6. Fisetin

Fisetin is a flavonoid found in many commonly consumed foods, such as strawberries [110], and has various biological properties that are beneficial in the treatment of AD. For instance, fisetin protects neurons from oxidative stress-induced death [111], and promotes neuronal differentiation [112,113]. In rats, Das et al. showed that fisetin (20 mg/kg, oral administration for 12 weeks) reduced lipid peroxides and preserved Na+/K+-ATPase activity which was found modified in the epileptic rats and also found to attenuate the seizure related cognitive dysfunctions [114]. In mice, Ashfaq et al. showed that Fisetin (20 mg/kg, intraperitoneally injected for 2 weeks) acts as a neuroprotector against Aβ1–42-induced neurotoxicity. It also induced increased levels of presynaptic (SNAP-25 and SYN) and postsynaptic (SNAP-23, PSD-95, p-GluR1 (Ser 845), pCAMKII (Thr 286) and p-CREB (Ser 133)) proteins which allows reversing synaptic dysfunction induced by Aβ1–42 [115].

### 3.7. Berberine

Berberine (BBR) is an isoquinoline alkaloid isolated from *C. chinensis.* Numerous preclinical and clinical studies have shown that BBR is beneficial in diabetes and increased peripheral and central cholinergic nervous system activity [116,117,118,119,120]. Lee et al. showed that berberine (20 mg/kg, i.p. for 14 days) decreased tumor necrosis factor-α, the expression of pro-inflammatory cytokines including interleukin-1β, and cyclooxygenase-2 mRNA in the hippocampus [116]. In rats, Hend et al. showed that berberine (50 mg/kg, orally) significantly improved cognitive behavior and provided protective effects against heavy metal-induced memory impairment [121].

## 4. Materials and Methods

**Figure 1:**
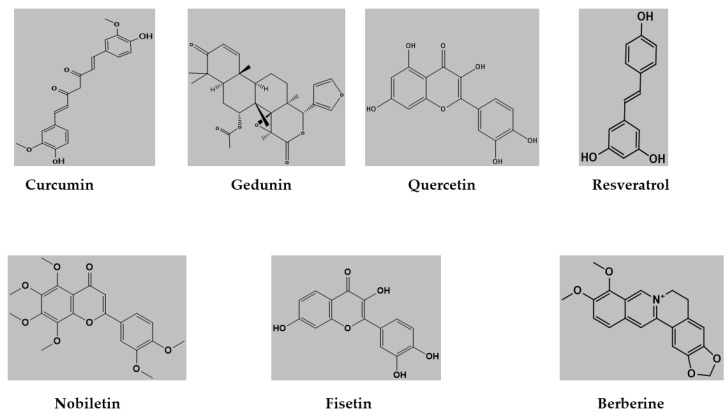
Structures of ligand molecules used for molecular docking.

The docking calculations were performed on curcumin ((1E,6E)-1,7-bis(4-hydroxy-3-methoxyphenyl) hepta-1,6-diene-3,5-dione), gedunin ([(1S,2R,4S,7S,8S,11R,12R,17R,19R)-7-(furan-3-yl)-1,8,12,16,16-pentamethyl-5,15-dioxo-3,6-dioxapentacyclo [9.8.0.02,4.02,8.012,17] nonadec-13-en-19-yl] acetate), quercetin (2-(3,4-dihydroxyphenyl)-3,5,7-trihydroxychromen-4-one), resveratrol (5- [(E)-2-(4-hydroxyphenyl)ethenyl]benzene-1,3-diol), nobiletin (2-(3,4-dimethoxyphenyl)-5,6,7,8-tetramethoxychromen-4-one), Fisetin (2-(3,4-dihydroxyphenyl)-3,7-dihydroxychromen-4-one), berberine (16,17-dimethoxy-5,7-dioxa-13azoniapentacyclo [11.8.0.02,10.04,8.015,20]henicosa-1(13),2,4(8),9,14,16,18,20-octaene) (Figure 1).

### 4.1. Protein Preparation

The crystal structure of human Acetylcholinesterase (AChE) with the Protein Data Bank (PDB) ID 4PQE was accessed from the website (Available online: www.rcsb.org/, accessed on 2 February 2023) [122]. The structure was prepared by removing all water molecules, and the Kollman charges and polar hydrogens were added to the protein using AutoDockTools (ADT) version 1.5.7 [123,124,125,126,127]. For the molecular docking process, a grid box with a point spacing of 0.375 Å and dimensions of 40 × 40 × 40 was created. The grid box was centered at coordinates x = 4.764, y = 65.53, z = 56.856 to encompass both the peripheral and active site regions of human AChE. The prepared structure was saved in a dockable pdbqt format to facilitate the subsequent molecular docking analysis.

### 4.2. Ligand Preparation

The structures of seven ligands, along with the standard inhibitor donepezil, were obtained in SDF format from the PubChem database (Available online: www.pubchem.ncbi.nlm.nih.gov/, accessed on the 2 February 2023) [128]. These ligand structures were then converted into the pdb format using PyMoL Molecular Graphics System (Version 2.5.3). Additionally, Autodock tools (ADT; version 1.5.7) were utilized to convert both the ligand molecules and the protein into the dockable pdbqt format [129,130,131].

### 4.3. Molecular Docking

Molecular docking is a crucial approach employed to explore the active site of proteins and unravel the intricate interactions between ligands and biological molecules [132]. To provide a comprehensive overview of the ligand–receptor interactions, Table 1 categorizes the compounds based on their affinity ratings, offering valuable insights into their binding properties. To visualize these interactions and generate informative graphics, the software Discovery Studio Visualizer v3.0 (BIOVIA, 2021) was utilized [133].

The process of molecular docking allows us to delve into the structural details of ligand binding and assess the strength of their interactions with the target receptor. By examining the binding affinity, we can discern the potential efficacy and selectivity of the ligands in comparison to conventional inhibitors. This information aids in understanding the molecular mechanisms underlying the therapeutic effects of the compounds and provides a basis for further investigations.

### 4.4. ADME Studies

To assess the drug-likeness of the seven phytochemical compounds, absorption, distribution, and metabolism (ADME) studies were conducted [134]. The ADME properties were determined using the Swiss online ADME web Tool [135,136]. Furthermore, the relationship between the calculated lipophilicity (WLOGP) and the polar surface area (TPSA) of the compounds was analyzed using GraphPad Prism software v8.0 (GraphPad Software, San Diego, CA, USA). This analysis allowed for the evaluation of the blood–brain barrier (BBB) properties of the compounds [137].

## 5. Results and Discussion

### 5.1. Molecular Docking Results

**Figure 2:**
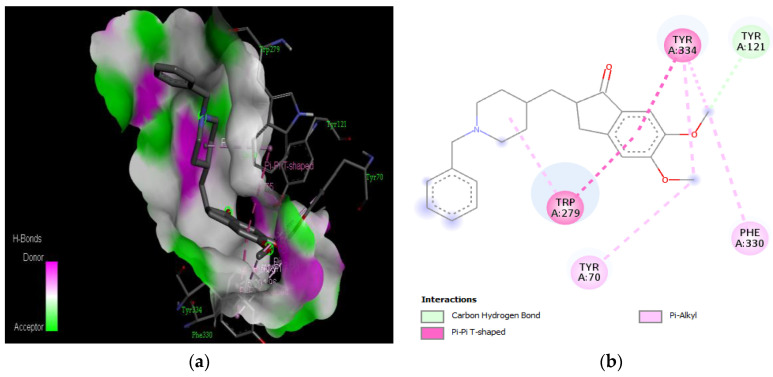
Active site of the acetylcholinesterase (AchE) with donepezil: (**a**) H-bond interaction between donepezil and (AchE); (**b**) two-dimensional view the interactions between the ligand and the amino acids of the receptor.

**Figure 3:**
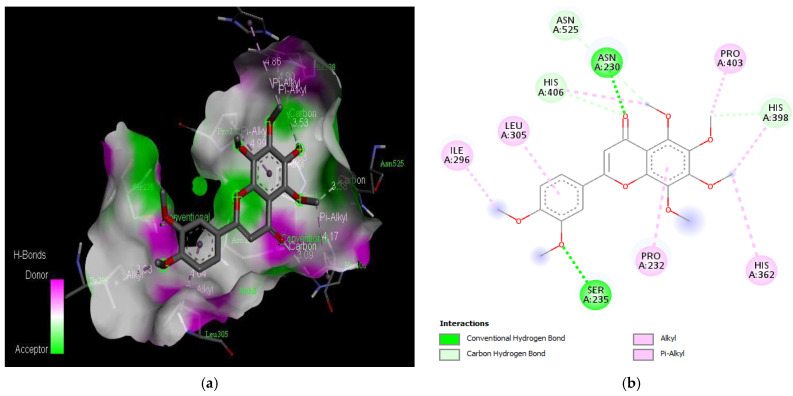
Active site of the acetylcholinesterase (AchE) with nobiletin: (**a**) H-bond interactions; (**b**) two-dimensional view the interactions between the ligand and the amino acids of the receptor.

The molecular docking results revealed that gedunin exhibited a higher binding affinity (−8.7 kcal/mol) for AChE compared to the standard AChE inhibitor (Figure 2), donepezil (−8.6 kcal/mol), as shown in Table 1. Following closely behind gedunin, the ligands fisetin, berberine, curcumin, quercetin, resveratrol, and nobiletin exhibited binding affinities of −8.2, −7.7, −7.5, −7.5, −7.0, and −6.9 kcal/mol, respectively, for AChE. Notably, nobiletin displayed the lowest binding affinity (−6.9 kcal/mol) among the studied ligands but interacted with crucial amino acid residues of AChE, as depicted in Figure 3. These findings align with previous studies indicating the inhibitory effect of nobiletin on AChE [138,139,140].

**Figure 4:**
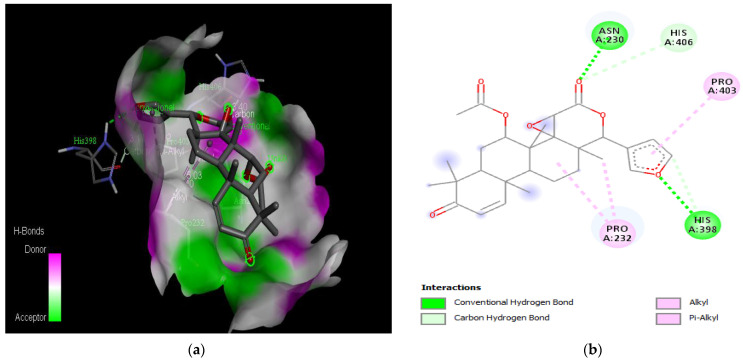
Active site of the acetylcholinesterase (AchE) with gedunin: (**a**) H-bond interactions; (**b**) two-dimensional view the interactions between the ligand and the amino acids of the receptor.

**Figure 5:**
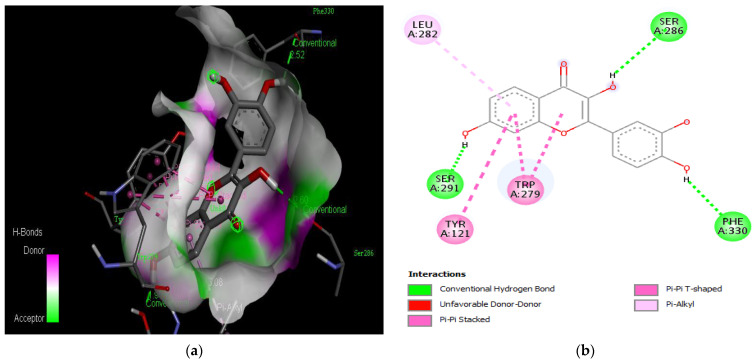
Active site of the acetylcholinesterase (AchE) with fisetin: (**a**) H-bond interactions; (**b**) two-dimensional view the interactions between the ligand and the amino acids of the receptor.

**Figure 6:**
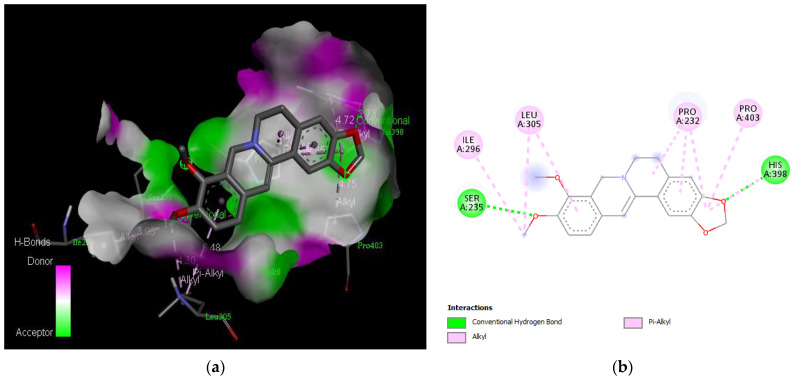
Active site of the acetylcholinesterase (AchE) with berberine: (**a**) H-bond interactions; (**b**) two-dimensional view the interactions between the ligand and the amino acids of the receptor.

**Figure 7:**
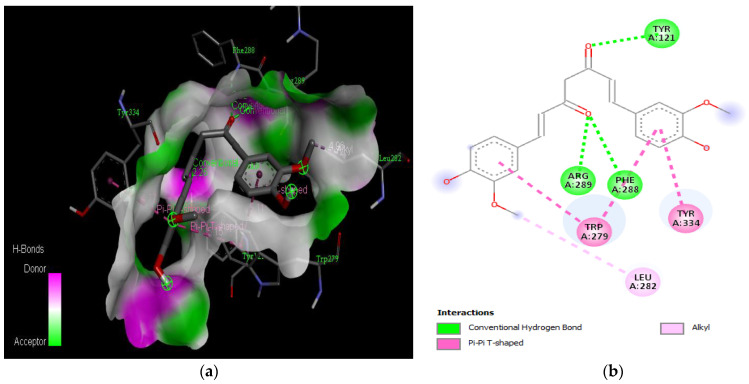
Active site of the acetylcholinesterase (AchE) with curcumin: (**a**) H-bond interactions; (**b**) two-dimensional view the interactions between the ligand and the amino acids of the receptor.

**Figure 8:**
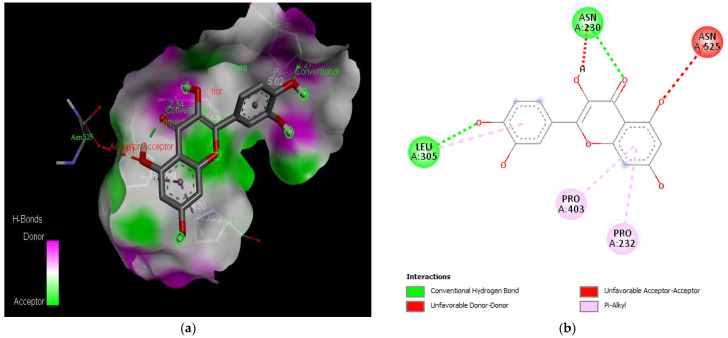
Active site of the acetylcholinesterase (AchE) with quercetin: (**a**) H-bond interactions; (**b**) two-dimensional view the interactions between the ligand and the amino acids of the receptor.

**Figure 9:**
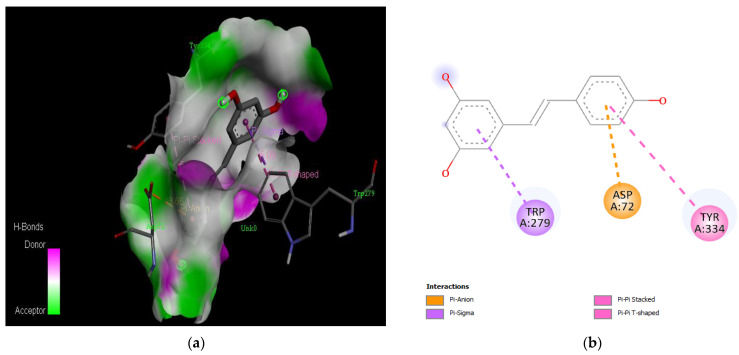
Active site of the acetylcholinesterase (AchE) with resveratrol: (**a**) H-bond interactions; (**b**) two-dimensional view the interactions between the ligand and the amino acids of the receptor.

Comparing the ligands to the standard inhibitor donepezil, gedunin, fisetin, berberine, and curcumin exhibited favorable affinities toward AChE (Figure 4, Figure 5, Figure 6 and Figure 7). Particularly, gedunin displayed the weakest binding energy among the ligands and even lower than the standard inhibitor, with a binding affinity of −8.7 kcal/mol. Furthermore, noteworthy interactions were observed between these four ligands and important amino acid residues at the active site of AChE [141]. For a better understanding of the interactions between these ligands and the active site of AChE, Figure 2, Figure 3, Figure 4, Figure 5, Figure 6, Figure 7, Figure 8 and Figure 9 present the 2D structures of AChE-ligand complexes, illustrating the hydrogen bond interactions.

Acetylcholinesterase (AChE) possesses a narrow gorge measuring about 20 Å, which consists of two main regions: the peripheral anionic site (PAS) located near the entrance and the catalytic active site (CAS) situated at the bottom [131]. The AChE structure contains several conserved aromatic amino acids, including Tyr 70, Asp 72, Trp 84, Gly 118, Gly 119, Tyr 121, Tyr 130, Ser 200, Ala 201, Trp 279, Phe 288, Phe 290, Glu 327, Phe 330, Phe 331, Tyr 334, and His 440 [142]. Inhibitors that target AChE hold promise for Alzheimer’s disease (AD) treatment, as they can bind to one or both of these binding sites [143].

The PAS of AChE is composed of amino acids Tyr 121, Trp 279, Tyr 70, Asp 72, and Tyr 334. It is responsible for cation-π interactions with the quaternary ammonium group of the substrate. The anionic site of AChE, comprising Trp 84, Tyr 130, Phe 330, and Phe 331, plays a role in inhibitor binding and interacts with the substrate. The proper orientation of acetylcholine within the gorge facilitates interactions with the anionic site [142]. In the case of gedunin, it forms multiple hydrogen bonds with key amino acid residues of AChE, specifically Asn 230 and His 398. Additionally, it engages in two pi-alkyl interactions with Pro 232, a pi-alkyl interaction with Pro 403, and a carbon–hydrogen bond interaction with His 406 (Figure 2b). Curcumin demonstrates two pi-pi T-shaped interactions with Trp 279, a pi-pi T-shaped interaction with Tyr 334, and a hydrogen bond interaction with Tyr 121, all of which involve important amino acid residues of the PAS (Figure 5b). Resveratrol exhibits a pi-sigma interaction with Trp 279 and a pi-pi T-shaped interaction with Tyr 334 (Figure 7b). Fisetin demonstrates a pi-pi stacked interaction with Tyr 121, two pi-pi T-shaped interactions with Trp 227, and a hydrogen bond interaction with Phe 330, which is a significant amino acid residue of the anionic site (AS) (Figure 3b).

### 5.2. Results of ADME Analysis

The findings of the ADME investigation on the seven compounds with high binding energy for acetylcholinesterase are summarized in Table 2. Notably, the results indicate that all seven compounds exhibit characteristics that make them suitable candidates for oral medication. Importantly, none of the compounds violated Lipinski’s rule, which implies that they possess favorable drug-like properties. Specifically, these compounds have a molecular weight of less than 500 Daltons, fewer than ten hydrogen bond acceptors, fewer than five hydrogen bond donors, and an octanol/water partition coefficient (MLogP) of less than five. Additionally, each of the seven compounds has a topological surface area (TPSA) smaller than 140 and fewer than ten rotatable bonds, complying with Veber’s criteria. These favorable ADME properties enhance the potential of these compounds as viable oral medications.

Lipinski’s rule of five is a well-established standard in the field of drug discovery, outlining a set of physicochemical properties that characterize orally active compounds. According to Lipinski, compounds suitable for oral drug development generally have no more than one breach of the following criteria: (1) Fewer than five hydrogen bond donors (oxygen or nitrogen atoms with one or more hydrogen atoms), (2) Fewer than 10 hydrogen bond acceptors (oxygen or nitrogen atoms), (3) Molecular mass less than 500 Daltons, and (4) Octanol-water partition coefficient log P (MLogP) not exceeding five [123,144]. The seven compounds being studied in this research adhere to Lipinski’s criteria, with no more than one violation each, and they exhibit notably strong negative binding affinities. Additionally, the compounds were evaluated based on Veber’s rule, which takes into account the number of rotatable bonds and the polar surface area [145]. Significantly, none of the molecules violated Veber’s criteria, further bolstering their potential as promising candidates for oral drug development.

**Figure 10:**
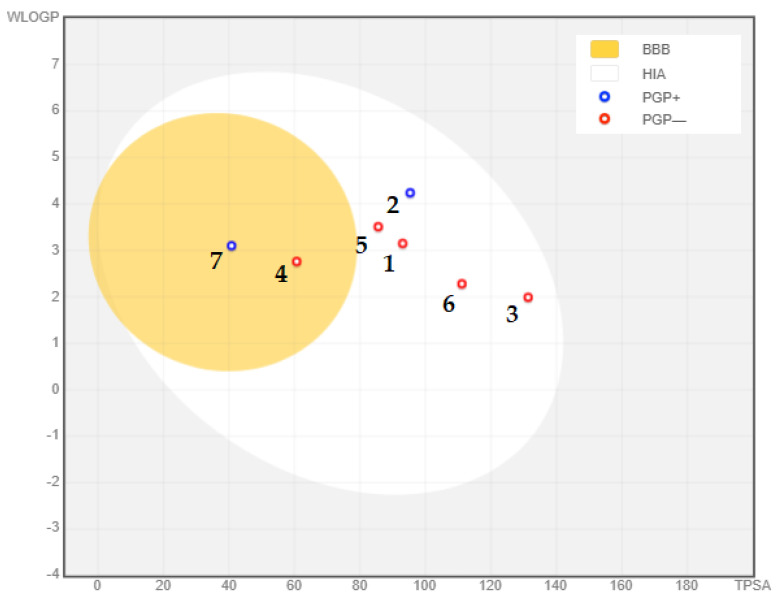
Blood–brain barrier properties of phytochemical compounds with significantly remarkable binding affinity to acetylcholinesterase: (**1**) curcumin; (**2**) gedunin; (**3**) quercetin; (**4**) fisetin; (**5**) resveratrol; (**6**) nobiletin; (**7**) berberine. PGP+: P-glycoprotein substrate; PGP-: non-substrate of P-glycoprotein.

Considering the compounds’ potential to enter the central nervous system (CNS), it is essential to assess their ability to cross the blood–brain barrier (BBB). Typically, compounds that are moderately lipophilic (WLogP 0.4–6.0) and have moderate polarity (TPSA < 79 Å2) can penetrate the BBB [135]. In Table 2, only resveratrol (TPSA = 60.69 Å2, WLogP = 2.76) and berberine (TPSA = 40.80 Å2, WLogP = 3.10) meet these criteria, indicating their potential to cross the BBB. Additionally, most of the analyzed compounds were determined to be non-substrates of P-glycoprotein (PGP-) within the yellow zone, except for gedunin (two) and berberine (seven). This is illustrated in Figure 10, where two compounds fall within the yellow region (indicating PGP- non-substrates), while the remaining five compounds (BBB-) are located outside the yellow region. The BBB serves as a vital barrier between the systemic circulation and the CNS, protecting the brain through both biochemical and physical barriers, including enzymatic activity and active efflux [135,146]. Overcoming the challenges posed by the BBB is critical in the development of effective CNS drugs, and the ability of the selected compounds to cross the BBB holds importance in Alzheimer’s disease treatment [147]. Overall, the adherence of the compounds to Lipinski’s rule of five, their compliance with Veber’s rule, and their potential to cross the BBB indicate their viability as potential oral drug candidates for the treatment of Alzheimer’s disease.

## 6. Conclusions

In recent decades, there has been a significant advancement in our understanding of psychotropic plants and their neuroprotective phytochemicals. These natural compounds have shown great potential in preclinical studies for the treatment of Alzheimer’s disease. However, it is crucial for patients to approach phytomedicines with caution, as not all natural substances are entirely harmless. While promising results have been obtained in animal models, therapeutic effectiveness and safety in humans cannot be assumed. Cholinesterase inhibition has emerged as an effective therapeutic strategy for neurodegenerative disorders, including Alzheimer’s disease. The findings from our study suggest that all seven compounds investigated have the potential to be considered as oral medication candidates. Notably, fisetin and berberine demonstrate reasonable binding affinity for acetylcholinesterase, but it is gedunin that exhibits the highest binding affinity compared to the standard inhibitor used. This highlights the potential of gedunin as a potent inhibitor of acetylcholinesterase. Moreover, resveratrol and berberine demonstrate the ability to cross the blood–brain barrier. This is an important characteristic as it enables the compounds to reach the central nervous system effectively. Therefore, all seven molecules identified in this study possess the capability of being orally administered, making them potentially more effective in the treatment of neurodegenerative diseases compared to the existing drug, donepezil. Overall, our findings contribute to the growing body of evidence supporting the potential of phytochemicals in the treatment of Alzheimer’s disease. However, further research and clinical studies are needed to validate their therapeutic effectiveness and safety in human patients.

## Figures and Tables

**Table 1:** Summary of molecular docking studies of phytochemical compounds against AChE.

Compound	Binding Affinity (Kcal/mol)	Interaction Site
Donepezil	−8.6	Tyr 70, Tyr 121, Trp 279, Phe 330, Tyr 334
Curcumin	−7.5	Tyr 121, Trp 279, Leu 282, Phe 288, Arg 289, Tyr 334
Gedunin	−8.7	Asn 230, Pro 232, His 398, Pro 403, His 406
Quercetin	−7.5	Asn 230, Pro 232, Leu 305, Pro 403, Asn 525
Resveratrol	−7.0	Asp 72, Trp 279, Tyr 334
Nobiletin	−6.9	Asn 230, Pro 232, Ser 235, Ile 296, Leu 305, His 362, His 398, Pro 403, His 406, Asn 525
Fisetin	−8.2	Tyr 121, Trp 279, Leu 282, Ser 286, Ser 291, Phe 330
Berberine	−7.7	Pro 232, Ser 235, Ile 296, Leu 305, His 398, Pro 403

**Table 2:** Physicochemical properties of phytochemical compounds with remarkable binding affinities against acetylcholinesterase.

S/N	Compounds	Molecular Weight	HBA	HBD	MLogP	Lipinski’s Violations	Rotatable Bonds	TPSA ${(Å}^{2})$	Veber’s Violation	WLogP	BBB Permeation
1	Curcumin	368.38	6	2	1.47	0	8	93.06	0	3.15	No
2	Gedunin	482.57	7	0	2.56	0	3	95.34	0	4.24	No
3	Quercetin	302.24	7	5	−0.56	0	1	131.36	0	1.99	No
4	Resveratrol	228.24	3	3	2.26	0	2	60.69	0	2.76	Yes
5	Nobiletin	402.39	8	0	0.34	0	7	85.59	0	3.51	No
6	Fisetin	286.24	6	4	−0.03	0	1	111.13	0	2.28	No
7	Berberine	336.36	4	0	2.19	0	2	40.80	0	3.10	Yes

WLogP: Lipophilicity; MLogP: Octanol/water partition coefficient; TPSA: Topological polar surface area.

## Data Availability

All the data supporting the findings of this study are included in this article.
